# Exploring athletic trainers’ experience and perceptions associated with a multifaceted approach to concussion management

**DOI:** 10.2217/cnc-2022-0010

**Published:** 2023-03-01

**Authors:** James Stavitz

**Affiliations:** 1 Athletic Training Education Department, Kean University, NJ 07083, USA

**Keywords:** head trauma, healthcare, public health, qualitative design, secondary school, sports medicine

## Abstract

**Aim:**

To explore high school athletic trainers’ experience with, and perceptions of, a multifaceted approach to concussion management.

**Participants:**

A total of 20 high school athletic trainers that are certified and licensed to practice (if their state requires a license to practice) participated in this study.

**Materials & methods:**

A general qualitative design with descriptive coding and saturation was met at 20 interviews.

**Results:**

Lack of standardization allows assessment, referral and return-to-play experiences to vary significantly; referral experiences vary pending upon athletic trainers ability to refer to a trusted and responsive physician; barriers include clearances from unqualified physicians; pressure from coaches, parents and students to return students to play; benefits include increased knowledge and awareness resulting in more effective care for students.

**Conclusion:**

Athletic trainers have varying experiences and perceptions regarding their approaches to concussion management. However, there were many notable similarities in the experiences, pressures, barriers and benefits when applying their concussion protocol.

No two concussions are the same, will present the same, nor should be managed in the same fashion. Thus, by using a multifaceted, multistep, approach to managing head trauma, athletic trainers (ATCs) are able to utilize more than one measure to assess trauma severity and return-to-play (RTP) status. It is extremely important that the evaluation and management of concussion takes a multifaceted approach to include a clinical evaluation, symptom checklist, neurocognitive assessment and balance assessment. ATCs frequently assess and treat concussions in high school (HS) athletes using this approach, and often serve as the primary treatment manager and decision-maker in clearing a HS athlete to RTP [1]. However, there is evidence that HS athletes are frequently cleared prematurely for RTP [2].

While athletic training education accreditation standards are quite exhaustive around the identification and differentiation of signs and symptoms of a concussion, there are no documentations that state athletic trainers ‘must’ follow an exact RTP protocol. However, a multifaceted approach to head trauma has become the gold standard and certain organizations, like the National Football League have set policies in place so that a particular procedure must be followed prior to letting an athlete return to the field. In most professional sports, if the procedures are not followed appropriately, a hefty fine will be imposed [3].

As athletic trainers are often the most knowledgeable person on the scene of an athletic injury, decision making may fall solely on their shoulders. Concussion management training for athletic trainers is essential. Athletic trainers’ adherence to concussion guidelines can prevent premature RTP, but there is evidence that HS athletes are frequently cleared for RTP while they are still symptomatic [4]. Identifying perceived challenges and barriers to the implementation of a multifaceted approach to concussion management (MFACM) would contribute to addressing and removing said challenges and barriers, and therefore ensuring the safety of HS athletes who have suffered concussions [5–7].

## Research question

What are HS ATCs experiences and perceptions associated with a MFACM?

### Methodology

In this study, the principal investigator (PI) explores HS ATC’s experience and perceptions associated with a MFACM through a general qualitative design. The PI recruited participants via National Athletic Trainers Association (NATA) membership email, snowball sampling and criterion sampling [8]. All subjects are board certified and licensed ATCs practicing in a HS setting and all participants report they are treating, or permitted to treat, concussion patients according to their school policy. The exclusion criteria are the contrary of the inclusion and membership in any population designated as vulnerable by the Institutional Review Board.

Data collection occurred in two steps: The first step included a letter of solicitation (LOS) with link to a prescreen survey with inclusion criteria. The LOS specifically details the inclusion criteria for potential participants. In order to ensure that all potential participants met the inclusion criteria, the PI developed a prescreening survey via Qualtrics™, which ask the participants to respond to questions specific to the inclusion status. This request is on the LOS as well as the link the Qualtrics survey. If participants responded ‘yes’ to all and are interested in participating in the survey, they were instructed to provide an email and submit the Qualtrics survey. The PI received and reviewed all completed surveys. For those individuals meeting the inclusion criteria, the PI contacted them via email to arrange for study consent and interview. Out of the 2500 ATCs that were contacted, collectively through all of the aforementioned recruitment strategies, 33 inclusion surveys were started, 25 were finished and 20 provided an email to reach out and schedule an interview.

The PI is specifically interested in taking a deeper dive into understanding what those who are practicing in a HS setting have in relation to one another. Qualitative generalization is a term used in a limited way in qualitative research, since the intent of this form of inquiry is not to generalize findings to individuals, sites or places outside of those under study. In fact, the value of qualitative research lies in the description and themes developed in the context of a specific area-of-study. Particularity, rather than generalizability, is the hallmark for good qualitative research [9].

**Figure 1.**
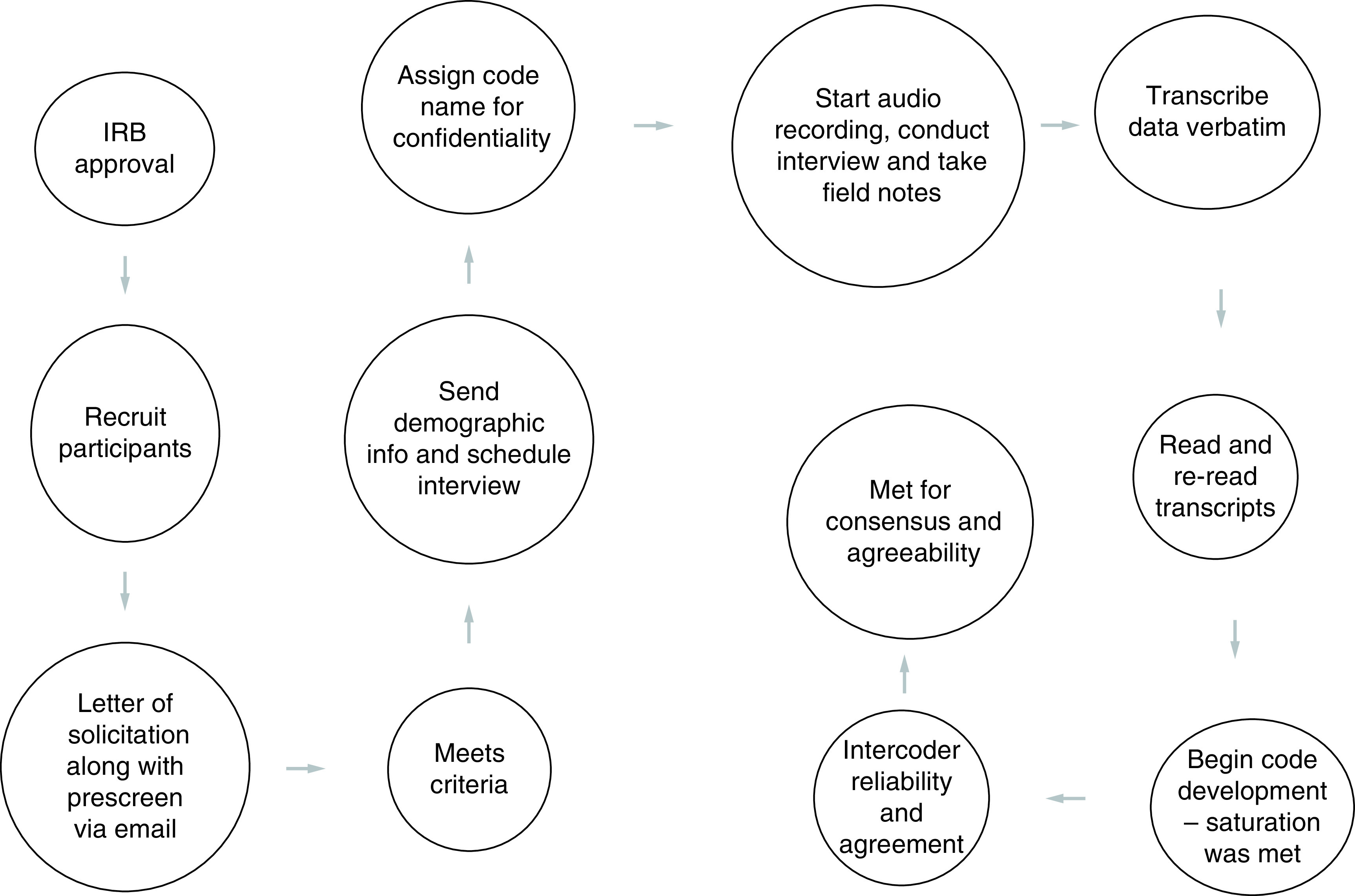
Interview process. IRB: Institutional review board.

**Figure 2.**
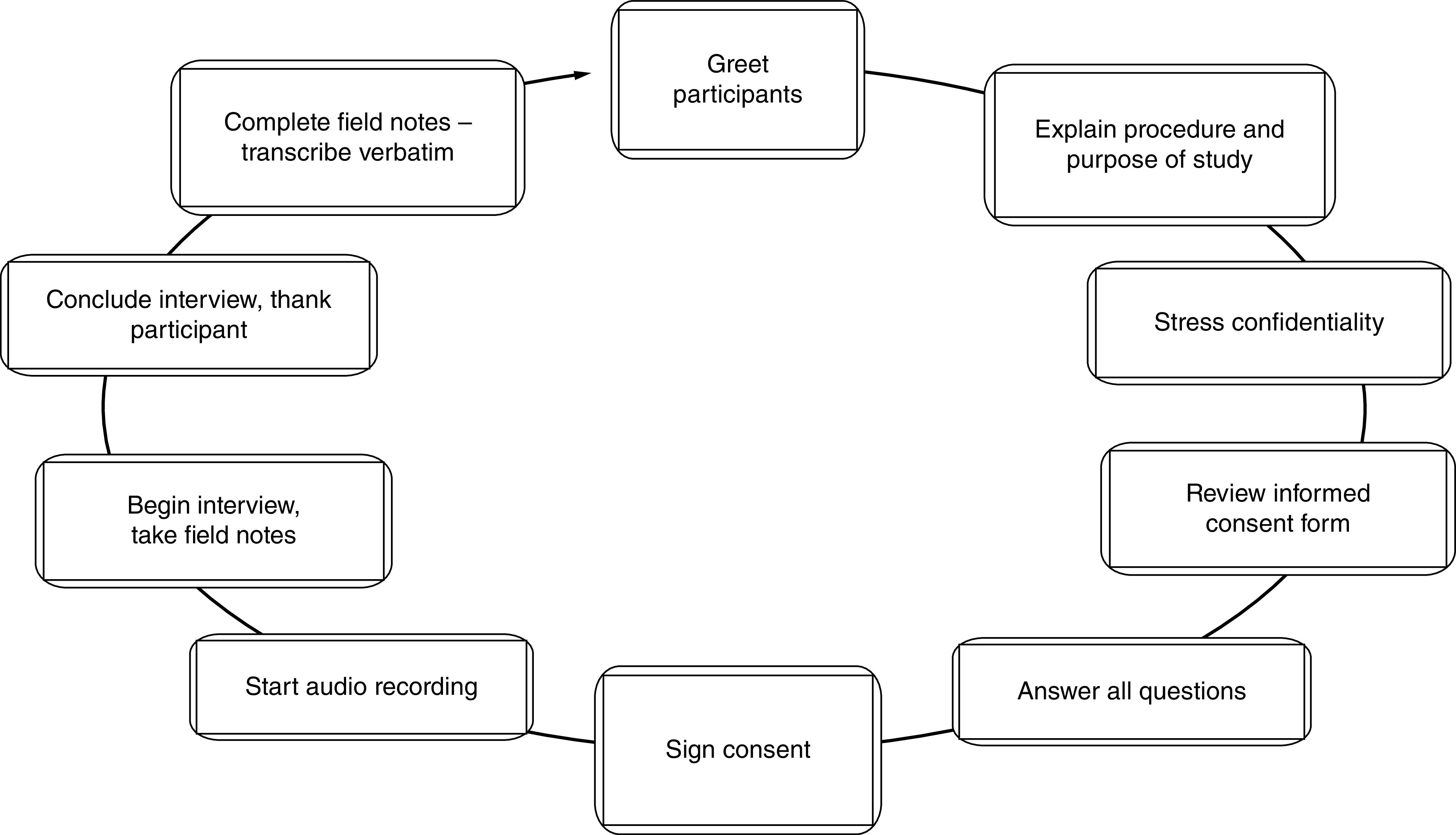
Data collection.

Data collection used a semi-structured interview approach (Figure 1) [10]. There are 12 interview questions, with probes, for further expansion (Table 1). The interviews were approximately 90 min for each interview and all have been recorded for post verbatim transcription (Figure 2).

**Table 1.** Themes.

Themes	Transcripts excerpts included (n)
1) Mental health issues affecting individual daily lives of AT	67
2) AT link mental health issues with stress	20
3) AT experience barriers to seeking help for mental health issues	43
4) AT have poor mental health help-seeking practices.	15

AT: Athletic trainers.

At the time of the interview, the PI briefly reviewed the purpose of the study to refresh the participant’s memory. Next, the PI reviewed the terms of informed consent form with the participant and asked if he or she has any questions. When all questions were answered to the participant’s satisfaction, the researcher requested that he or she print and sign the emailed informed consent form and email a scan or photo of the signed document. Next, the PI asked for the participant’s consent to turn on the audio-recorder. Once consent was given, the audio-recorder was turned on.

Guiding the interview was a protocol consisting of open-ended questions formulated to address the research question (RQ). If the participant answers the scripted questions too briefly, too vaguely or otherwise without providing adequate data, the PI asked probing and follow-up questions to elicit more information. In a general qualitative study, it is desirable for data to include rich and detailed accounts of participants’ perceptions and lived experiences [11]. Therefore, the PI attempted to elicit as much description and detail from participants as the interview duration allowed. After the PI asked all of the scripted questions, the PI asked the participant if he or she had anything he or she would like to add if there were any further questions or concerns.

When the PI answered any further questions from the participant, the PI requested the participant’s permission to email him or her the transcript of the interview for him or her to review and correct. Next, the PI ensured that the participant has the appropriate contact information in case any issues or questions related to the study arose. At last, the PI thanked the participant for his or her time and concluded the interview. Data collection were carried out in uniform for all regions in which the participants are located to ensure consistency and reliability [12].

### Data analysis

**Figure 3.**
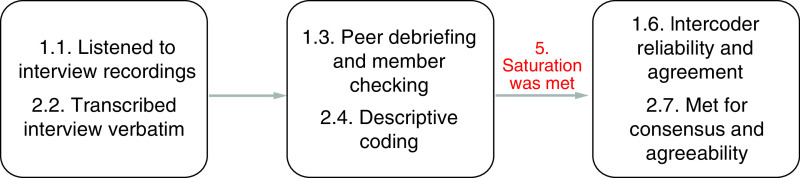
Data analysis.

Data analysis (Figure 3) began with reading the data to gain familiarity as well as transcribing the recorded interviews, verbatim, to assure accuracy. The PI, personally, coded the transcribed interview data for analysis instead of using a computer software. Once initial codes were established and common categories were generated, themes were created and two outside individuals (a certified and licensed ATC and an orthopedic surgeon, fellowship trained in sports medicine) were used to read over select interviews and codes for agreement as well as validity and reliability [13]. After all codes were created, themes were developed to interpret the findings and address the RQ. At the same time, the themes and the data were analyzed using the theoretical frame that the PI had selected, Knowledge to Action Framework, in which scholars recommends five to six themes be developed [14].

The PI achieved credibility by conducting peer debriefing to provide an external check on the research process, as well as member checking to test the findings and interpretations with the participants. The PI established trustworthiness and dependability via audit trails. Confirmability was established when credibility, transferability and dependability were all achieved [15–18].

## Results

### Demographics

Based on gender, 60% of the participants were males, while 40% were females. The NATA reports that 45% of the members are males, so this number is higher than the national breakdown of certified athletic trainers. Based on experience, 20% of the participants had less than 5 years of experience, 30% had 6–10 years of experience, 10% had 11–20 years of experience, 25% had 21–30 years of experience and 15% had greater than 30 years of experience. Based on level of education, 80% of participants received their Bachelor’s degree in Athletic Training, 13% of participants received their Bachelor’s degree in Exercise Science or Exercise Physiology and 7% of participants received their Bachelor’s degree in Health and Physical Education. Participants were also asked their highest degree they held. In this study, 60% of participants held advanced degrees, one of them being a terminal degree and the others being Master’s degrees. The remaining participants held their highest level of education at the Bachelor’s level.

To add, participants’ identities have been kept confidential and interview transcripts have been de-identified through the removal of participants’ names and any other identifying details, including but not limited to the name of the employing HS and its specific geographic location. However, 60% of the participants came from the Northeastern region of the US, 15% came from the Southeastern region, 10% came from the Midwest and 5% came from the South, Southwest and Northwest, respectively.

### Themes

Five major themes emerged during data analysis to address the RQ (Table 2).

**Table 2.** Interview questions.

1. Tell me a little bit about yourself.
2. Why you decided to become an athletic trainer?
3. What you know about mental health?
4. As an athletic training student, describe a time, if at all, that you personally experienced issues with mental health.
5. As an athletic training student, describe a time, if at all, that you had to seek professional support to help you deal with a personal mental health issue.
6. As an athletic training student, what is the biggest challenge you face when dealing with personal mental health?
7. As an athletic training student, what are some barriers, if any, that you may experience when dealing with personal mental health?
8. As an athletic training student, what are some pressures, if any, that you may face when dealing with personal mental health?
9. As an athletic training student, what are some benefits to dealing with your personal mental health issue?
10. If you can remember, has your personal mental health improved or regressed since becoming an athletic training student?
11. As an athletic training, what are some ways you can improve your mental health?
12. Are there any other questions or comments you would like to make regarding athletic training education, mental health, mental health as a student or anything at all?

### Theme 1: Lack of standardization allows assessment, referral & RTP experiences to vary significantly

When participants described their current approaches to concussion management in general terms, their descriptions were consistent. However, when participants described specific triggering conditions for steps in the procedure, their responses varied significantly. Participants attributed uncertainties about best practices to the lack of a mandated, standardized approach to concussion management (Table 3).

**Table 3.** Theme 1 codes.

Code	Participants contributing (n)	Transcript excerpts included (n)
Varying perceptions of state guidelines	17	19
Inconsistent referral policies	15	19
Inconsistent assessment practices	16	18
Inconsistent return-to-play policies	15	15

All participants reported that their current procedure for assessing student athletes for concussion involved an initial, on-field assessment using a standardized instrument such as the state-issued form. However, comparison of responses across participants indicated significant inconsistencies in their knowledge and implementation of practices. First, participants differed in the approaches they used to detect concussion symptoms during the on-field assessment, and they expressed differing levels of confidence in the instruments they currently used.

Similar inconsistencies emerged from participants’ responses describing when a physician referral was necessary or appropriate for students who exhibited symptoms of concussion. Some participants believed a physician referral in such cases was an immediate legal requirement, because they had the misconception that the policy and guidance for prevention and treatment of sports related concussions and head injuries in their particular region was a prescriptive statute rather than a recommendation. However, not all participants perceived a physician referral as mandatory or even advisable for students with mild or moderate concussion symptoms immediately following an impact.

### Theme 2: Referral experiences vary pending up ATCs ability to refer to a trusted & responsive physician

Participants reported that they were satisfied with their current referral procedures when they exercised significant discretion over the doctors to whom students were referred. Their discretion allowed them to send students to concussion specialists of proven competence whose assessments of students’ conditions were perceived as trustworthy. Participants’ experiences of referral procedures were either less positive or negative when there were significant delays before students could be seen by a physician and when the participant did not have sufficient discretion to ensure the student was evaluated by a trusted specialist. With that said, when participants expressed dissatisfaction with their experiences of physician referrals, they attributed it to parents’ declining to take the student athlete to a trusted provider (Table 4).

**Table 4.** Theme 2 codes.

Code	Participants contributing (n)	Transcript excerpts included (n)
Referral policy is satisfactory, but parents may ignore advice	15	14
Referral policy and practice allow a highly satisfactory level of AT discretion	15	10
Referrals are a frustrating ‘free-for-all’	1	1

### Theme 3: Barriers include clearances from unqualified physicians

Participants reported that barriers associated with a MFACM included the ability under their state law for physicians whom the ATC deem unqualified to assess concussions to issue clearances for student athletes to RTP. Participants reported that some students were cleared to RTP by physicians who, according to the athletic trainer, were not qualified to accurately assess concussion symptoms. Yet, the willingness of misinformed parents to rely on clearances from unqualified physicians was increased by limitations in insurance coverage that made assessments by specialists prohibitively costly (Table 5).

**Table 5.** Theme 3 codes.

Code	Participants contributing (n)	Transcript excerpts included (n)
Challenges of working with non specialist physicians	16	20
Parents have ultimate control over the choice of physician	13	14
Parents lack of knowledge about appropriate assessment	12	13
Limitations to health insurance coverage	14	19

Limitations on health insurance coverage was a code that helped create Theme 3: Barriers include clearances from unqualified physicians. Clearances from unqualified physicians had the potential to be inaccurate and to result in premature RTP and danger to the student. Parents who were uninformed about best practices for assessment, especially when limitations in the child’s insurance coverage made qualified specialists costly to access, were perceived as more likely to rely on potentially inaccurate clearances from unqualified physicians. While the transcript excerpts did mention ‘insurance’ numerous times, there was not enough evidence there to make it its own separate theme. However, it did warrant a code to help create the theme.

Theme 2 and Theme 3 both note challenges regarding physician referrals. As noted in Table 4 and Table 5, the high number of codes that created each theme and the high number of transcript excerpts included in each code gave cause to create two separate themes.

### Theme 4: Pressure from coaches, parents & students to return students to play

Participants reported that when assisting with concussion management they experienced pressure from coaches, parents and/or the students themselves to return students to play as quickly as possible. However, two participants provided discrepant data indicating that they experienced no pressure (Table 6).

**Table 6.** Theme 4 codes.

Code	Participants contributing (n)	Transcript excerpts included (n)
Coaches who want athletes playing	15	16
Pushback from students and parents	17	17
Discrepant data: no pressure	2	2

### Theme 5: Benefits include increased knowledge & awareness resulting in more effective care for students

The majority of the participants indicated that a benefit of a multifaceted approach was to educate ATCs about and raise their awareness of concussion and concussion management. The result of participants’ increased knowledge and awareness was more effective care for students. No participants provided discrepant data indicating that benefits did not include increased knowledge and awareness, or that increased knowledge and awareness did not result in better care for students (Table 7).

**Table 7.** Theme 5 codes.

Code	Participants contributing (n)	Transcript excerpts included (n)
Increased knowledge results in better practices for treating	17	20

Participants reported that implementation of a MFACM increased their own knowledge and awareness of concussion, its signs and symptoms and its appropriate management. This increased knowledge and awareness contributed to participants ability to detect signs and symptoms of concussion and to contribute to providing better care for student athletes with concussion.

### Summary

Five major themes emerged during data analysis to address the RQs. Codes were grouped to form the themes. The presentation of each theme also includes direct quotations from the data as evidence for the findings so the reader can independently confirm them (Table 8). The inclusion of direct quotations will also assist future researchers in assessing transferability by providing a rich description of the data in participants own words.

**Table 8.** Robust quotes used to make themes.

Theme	Quote
Theme 1	P1: “What we're using (for on-field assessments) is a written document that has been provided by the State of New Jersey. It is a one-page sheet that describes the signs and symptoms of a concussion and the appropriate actions that should be taken should the student athlete exhibit any of those signs or symptoms. Once those signs and symptoms are presented to a school official, the next step is to run it through the athletic trainer, who describes the policy to the parents and (informs the parents) that the child needs to be evaluated by a physician trained in concussion management.” P9: “Unfortunately, the SCAT is a great resource, but I feel like most kids even with a concussion can do very well on the SCAT form.” P2: “I will use an on-field evaluation such as the SCAT … Most athletes know how to fake (not having a concussion) till they make it, (but) you cannot fake your cognitive or your motor (function), so it is important that it is not just, ‘Do you have blurry vision? No? Okay, you are ready, you can go back.’” P10: “From an assessment standpoint, you really have to use a multifaceted approach. You cannot just rely on if they have a headache or not. There are just so many other things that can be affected. So, looking at the sense of balance, talking to the parents, talking to people who see (the injured athlete) on a day-to-day basis. Giving them some set time as well, if they do not have a headache five or ten min after the injury, but 24 h post-, now it is a different story. So, you definitely have to make sure that your diligence and making sure that you are, you know, you take a multifaceted approach.” P11: “By law, (students athletes exhibiting signs of concussion) need to be seen and cleared by a physician who is trained in the evaluation and management of head injury and concussion.” P6: “That is in the law: if an athlete has signs and symptoms of concussion, they need to be referred to a concussion specialist.” P1: “Our state instituted a law for the management of concussions, and our (school) policy follows the standards of that law.” P8: “We follow the state guidelines for concussion management, that (students) have to get cleared by a doctor. Then we do follow the 7 day symptom free (guideline) before they can do the return-to-play protocol.” P7: “We wait 24 h and see how they feel. If they feel fine in less than 24 h and a SCAT was totally clear or almost, like almost perfect 29 and 30, somewhere around there. As opposed to the patient who comes in the next day and says, ‘I feel worse or I am still hurt, I still have a headache.’ Then we will call the parents and say, ‘Okay, he is not going to be able to play until he sees a doctor.’ Then they will see the doctor, and they will start the protocol.” P7: “It is usually 24 h symptom-free, then return to some moderate activity. Maybe the first and second day, treadmill and then a couple of days of practice. The first day (of practice), a little less vigorous, less contact and then the second day of practice full. Then a final doctor’s clearance, with final review of their post injury impact test from the doctor.” P5: “Some students exhibit symptoms right away and we begin to facilitate a physician visit. Others we monitor overnight and into the next day to see if and how symptoms progress and then make a decision at that time.” P2: “The policy is set forth by whatever policy company creates policies for the school, based off of Zurich statement … our team doctor is the one that can amend or waive some of the policy.” P2: “We have to defer to the parent. Ultimately, it is their decision. I give the sheet that has the signs and symptoms … I try to give them the information I have myself. I give them my opinion.” P4: “(The procedure for referrals) was never specifically just spelled out. I just always remembered, even at the college level, it is between you and the doctor to kind of get (the athlete) back in play. I am sorry, I do not think I have ever heard someone specifically say a ‘multifaceted approach.’” P8: “I wish the state would be more specific with the laws and following this 7-day protocol, because it is only a guideline.” P8: “(The guidelines) say that you need to be cleared by a doctor, but they are not specific on what kind of doctor.”
Theme 2	P1: “I think we are lucky in our area that there are about ten physicians that do a really great job … between those ten physicians, it never takes more than a couple of days to get an appointment.” P5: “I am very fortunate to have a great group of physicians that work closely with our student athletes. Referral is often very quick and painless.” P6: “I absolutely love it … the concussion specialists, those guys are phenomenal … If anything happens or they evaluate somebody, they call me up right away.” P4: “I do not love it.” P4: “Parents here already have doctors that they like, or, ‘Oh, I heard so-and-so went to this doctor and he cleared (the student to return to play) in 2 days, so we are going to send our daughter there.’ So, that part I do not like, because it is kind of a free-for-all. I call the parent and let them know, ‘Hey, your daughter has a possible concussion. She needs to get evaluated by a doctor, a concussion specialist. Here is a place that I recommend. Here is the information.’ Then whether they go there or not, it is a crapshoot.” P2: “It is tough, because a lot of our athletes are just going to be going to a general practitioner or a walk-in … some of our student athletes have issues with getting to neurologists.” P3: “It depends on the doctors that (the students) go to.” P9: “Unfortunately, some of the parents and their kids like to try to get around (the referral requirement). I have had someone cleared by a foot doctor. I had someone cleared by a gynecologist.”
Theme 3	P3: “I have had doctors say, ‘Well, if they did not throw up, it is not a concussion.’ I was like, ‘that is not how it works.’” P8: “(Some doctors) won’t even recognize an injury as a concussion, because their justification is, ‘He did not lose consciousness,’ or, ‘He did not vomit.’” P8: “[Uninformed doctors will] return a kid back to play before their symptoms are resolved.” P3: “It is frustrating to know that the majority of doctors still don't know the updated information when it comes to treating concussion or knowing what a concussion is.” P8: “If the doctor is trained in concussions, I feel fairly secure and safe with that and I do not have a problem returning kids to play.” P1: “Because the law does not specifically speak to what does it means to be trained in concussion management, the family doctor could just turn around and say, ‘I read an article, so I know what I am doing.’” P1: “The most difficult challenge is convincing the parents they should go to a small subset of physicians in our area who really know what they are doing in concussion management and not just go to the family doctor.” P4: “A barrier would be just educating the parents on what RTP means. They hear RTP, and they are like, ‘Great, (the student’s) gonna quit play for 2 hours,’ whereas that is not a RTP.” P2: “A parent may come back with a clearance from imaging and think that that is okay … (but) the CAT scan’s not going to show a concussion.” P6: “The barriers are, I know the crappy doctors from the good doctors, and when a parent says, ‘I am gonna take him over here (to an unqualified doctor),’ there is nothing I can do, because I am not allowed to say, ‘We need to take him (to a qualified doctor instead).’ The parent’s choice is to take (the student) wherever their insurance covers and wherever the parent wants to drive to get a recommendation.” P8: “(A barrier has) been insurance issues. If I try to get (students) to a concussion center or a department that specializes in concussion, their insurance may not cover it.” P9: “We have some income issues in our area. So sometimes insurance is an issue … It is a little struggle getting the kids to the doctor in a timely manner, because the parents do not have insurance.”
Theme 4	P2: “Overbearing coach … wants to put (the student) right back in (play), but you need to let these things simmer a little bit to see if there is the progression of an injury.” P7: “There was a lot of pressure with coaches (saying), ‘So Johnny came out and they had their head hurt, but that is football.’” P8: “The coaches tend to pressure me. Like, ‘Oh, there is no concussion.’ They will deny the injury or the severity of the concussion, like, ‘Oh, he just got dinged,’ or ‘They are faking it.’” P1: “When I make that initial determination that this individual has enough signs and symptoms of a concussion that they need to be placed into the concussion protocol, the pushback that I get (is) from the parents, because they know that their child, without question, is going to be out of activity for what seems to them to be an eternity, that it is going to be 1, 2 and 3 weeks.” P9: “The coaches and the parents are on you to get (concussed athletes) cleared as quick as possible: ‘Why do they have to use a protocol, why is it this, why is it that?’ They always question it.” P11: “The athlete and the parents and the coach, they want to get (the students back into play) sooner rather than later.” P11: “Lack of education on all parties fronts … not understanding RTP.” P5: “I make sure our parent body understands the protocols that are in place and that there are no exceptions to these protocols.” P6: While you need to get him back and he is our top player, it is just not going to happen. We are going to follow the state law and that is it.”
Theme 5	P11: “A multifaceted tool that we now have to use, from the comprehensive exam of the SCAT 5 with the use of impact and neurocognitive testing, we have a lot of options to better help our patients.” P5: “We are extremely successful in the management of concussions, and I believe this is due to a multifaceted approach.” P5: “The main benefit I see is that our students receive the best possible care and are healed before resuming activity.” P1: “The educational piece (of a multifaceted approach) has been the biggest thing. Before we started this policy, I saw two, three, maybe four concussions a year … (Since) the 1st year we instituted the education … we have averaged between 25 and 30 concussions a year … What it is telling me is for 8 or 10 years prior to the policy, there were still (laughs), 25 kids a year getting a concussion and I was only seeing two or three or four of them.” P7: “I think it is much better than when I first was dealing with concussions, 20 years ago. It is safer for the patient. We have a specific plan in place that seems to protect the patient from anything getting worse.” P10: “I think it is satisfying because, I think, we are doing whatever we can (in using a multifaceted approach) … to improve on what we can offer and get to our athletes.”

RTP: Return-to-play; SCAT: Sport Concussion Assessment Tool.

## Discussion

### Theme 1: Lack of standardization allows assessment, referral & RTP experiences to vary significantly

The study finding revealed that, though participants described their approaches to concussion management in consistent terms, the descriptions of events, which led to concussion management protocols, varied greatly. An example of varying protocols includes the school protocol for whether a student should be referred to a physician.

The findings suggest that concussion guidelines were guidelines rather than laws or consistent procedures, which results in some students returning-to-play differently from others. This finding supports and extends literature. According to experts, concussion management is critical to the long-term wellness of young athletes that should be thoroughly assessed for concussive symptoms [1–3]. All of the participants discussed concussion assessment following head injuries, which supports the researcher’s findings that concussion assessment is vital.

### Theme 2: Referral experiences vary pending up ATCs ability to refer to a trusted & responsive physician

The study results suggested that ATCs perceived school referral policies to be positive when the trainers had the ability to refer students to trusted physicians. Additionally, ATCs experienced the process more positively when students could be assessed in a timely manner. For participants who had a network of concussion specialists who they trust to deliver timely and accurate assessments, the participants perceived the referral policy positively.

Expert results suggest that, as there is no definitive protocol for concussion assessment, a large portion of the assessment is left up to the skills and experiences of the physician [4]. This suggests there may be substantial importance in concussions being assessed by specialists, rather than general physicians or physicians bellowing to unrelated specialties. P3 stated that some of their students will just go to a general practitioner, as obtaining an appointment with a specialist is challenging.

### Theme 3: Barriers include clearances from unqualified physicians

The study findings revealed that participants perceived clearances from unqualified physicians to be barriers to the school’s successful implementation of the protocols. The participants reported that students were often cleared to play by physicians who lacked demonstrable knowledge of concussion diagnosis and management. These results suggest that the doctors who are asked to participate in portions of the assessments, typically by parents who choose the physician, may not be capable of complying with the guidelines based on the lack of information.

Similar to Theme 2, the findings of theme 3 support and extend literature. A recent study found that there are no clear protocols for diagnosing and assessing concussions [6]. Due to the large degree of variability in assessment tools, substantial physician judgement is necessitated.

### Theme 4: Pressure from coaches, parents & students to return students to play

The findings related to Theme 4 suggest that parents, coaches and students often want to RTP prior to the completion of the assessments, which can result in breaks of protocol. Roughly half of the participants reported that they experience pressure from coaches to return athletes to play following a concussion. This is suggesting a high number of coaches who are eager for athletes to RTP prior to the completion of the concussion protocols. Parents and students may echo this pressure.

These findings confirm recent literature on ATCs and RTP practices [5]. In a study of RTP practices, experts found that ATCs report a lower quality-of-life and job satisfaction due to pressure to clear students for athletic play before their professional judgement suggests they should be cleared [19].

### Theme 5: Benefits include increased knowledge & awareness resulting in more effective care for students

The study results suggested that ATCs perceive the MFACM protocols to result in increased knowledge and awareness, which results in more effective care for student athletes. This finding extends current literature on the MFACM. This research assessed concussion treatment protocols for student athletes. The study results indicated that roughly 47% of studied schools had a policy for concussion assessment and management in student athletes [4,5].

### Implications for practice

This study showed that the participants had significant varying experiences and perceptions regarding their multifaceted approaches to concussion management. Yet, notable similarities in the experiences, pressures, barriers and benefits were observed when applying their concussion protocol. There is little evidence looking at the experience and the perceptions of the individuals who are treating these injuries, especially at the HS level.

ATCs must not only identify and acknowledge these experiences and perceptions, but we must seek to develop strategies and initiatives through continuing education opportunities, position statements and clinical practice guidelines to help guide athletic training practice and ultimately improve the equity of care in HSs, specific to the management of concussion.

### Study limitations

One limitation of this study will be its reliance on the honesty and accuracy of participants reports of their perceptions and experiences. A second limitation of this study is that it cannot be known whether factors irrelevant to the study are influencing participants responses at the time of the interview. Another limitation is the sampling being used. This sampling is not random and therefore may not be fully representative of the entire population and because of the sampling; the results will not be generalizable to all ATCs. There were more males than females that participated in this study and the majority of the participants were from the Northeastern part of the US. To add, the participants in the study are currently practicing in various states throughout the country. While all of the participants are permitted to treat head trauma, certain states vary in their use of athletic trainers and their RTP policies.

### Suggestions for further research

Future research should consider whether general practitioners or unrelated specialists are able to accurately diagnose, assess and provide treatment protocols for potentially concussed student athletes. Second, the study revealed that ATCs were pressured into allowing students to RTP by coaches, parents and students. This pressure could negatively influence student outcomes by allowing students to play before they are medically fit. Future research should focus on understanding how athletic and education institutions could manage pressure from various source to return students to play prematurely.

## Conclusion

Robust quotes were used to This study helped show that there are benefits to using a multifaceted approach when managing a concussion. Participants in this study found that the MFACM provides more options for RTP assessments, helps ATCs provide better patient care and shows clinicians to be more confident in their concussive knowledge. Focusing on the benefits of the MFACM may help improve experiences, improve perceptions and increase knowledge on concussion management protocols. By supporting the uptake of knowledge, we can help address best practice for knowledge translation of clinical practice guidelines in the athletic training profession.

**Summary points**

- Lack of standardization allows assessment, referral and return-to-play experiences to vary significantly.
- Referral experiences vary pending upon athletic trainers ability to refer to a trusted and responsive physician.
- Barriers include clearances from unqualified physician.
- Pressure from coaches, parents and students to return students to play.
- Benefits include increased knowledge and awareness resulting in more effective care for students.
- There are benefits to using a multifaceted approach when managing a concussion.
- Participants in this study found that the multifaceted approach provides more options for return-to-play assessments.
- A multifaceted approach helps athletic trainers provide better patient care.
- A multifaceted approach shows clinicians to be more confident in their concussive knowledge.
- Focusing on the benefits of the multifaceted approach may help improve experiences, improve perceptions.
